# Clinical Workflow of Spine Stereotactic Radiotherapy and Radiosurgery: Insights from a Single-Institution Physics Perspective

**DOI:** 10.3390/cancers18030353

**Published:** 2026-01-23

**Authors:** Dennis Mackin, Gizem Cifter, Yana Zlateva, Jihong Wang, Yao Ding, Muhammad Shafiq ul Hassan, Zhiheng Wang, Parmeswaran Diagaradjane, Fada Guan, Travis C. Salzillo, Shane Krafft, Jing Li, Martin C. Tom, Amol J. Ghia, Tina Marie Briere

**Affiliations:** 1 Department of Radiation Physics, The University of Texas MD Anderson Cancer Center, Houston, TX 77030, USA; 2 Department of CNS Radiation Oncology, The University of Texas MD Anderson Cancer Center, Houston, TX 77030, USA

**Keywords:** spine stereotactic radiosurgery, spine stereotactic body radiotherapy, immobilization, SBRT immobilization, spinal metastases

## Abstract

Spine stereotactic radiotherapy and radiosurgery (SSRS) techniques, encompassing both fractionated stereotactic treatments and single-fraction radiosurgery, are widely used for the management of spinal metastases due to their ability to deliver highly conformal radiation while limiting dose to adjacent critical structures. Clinical outcomes following SSRS, including durable local control and acceptable toxicity, have been reported previously in multiple institutional series. In this manuscript, we describe the clinical workflow used to deliver SSRS at a high-volume academic center, with emphasis on the medical physics processes that support routine clinical practice. Key elements of the workflow include patient selection, treatment region-specific immobilization, CT and MRI-based simulation, treatment planning, patient-specific quality assurance, and image-guided treatment delivery. Rather than presenting new outcome data, this work provides a descriptive overview of how established SSRS techniques are integrated into day-to-day clinical care.

## Simple Summary

Spine stereotactic radiotherapy and radiosurgery (SSRS) is a precise form of radiation therapy used to treat cancer involving the spine, delivering high doses to tumors while limiting exposure to nearby critical structures. This manuscript describes the clinical workflow used to deliver SSRS at our institution, with attention to the medical physics processes that support routine clinical practice. Key elements include patient selection, treatment region-specific immobilization, CT and MRI-based imaging, treatment planning, patient-specific quality assurance, and image-guided treatment delivery, providing an overview of how SSRS is implemented in day-to-day clinical care.

## 1. Introduction

Spine stereotactic radiotherapy and radiosurgery (SSRS), encompassing both fractionated stereotactic treatments and single-fraction radiosurgery, has emerged as the preferred treatment modality for spinal metastases, offering superior tumor control and pain relief compared to conventionally fractionated radiation therapy [1,2,3]. Since initiating SSRS at our institution in 2002, we have conducted multiple studies demonstrating its durable local control and favorable toxicity profile [4,5,6,7,8,9,10,11,12,13,14]. Additionally, SSRS has been shown to be resource-efficient relative to conventional radiation therapy [15]. Reflecting this growing body of evidence and clinical acceptance, the number of patients receiving SSRS at our center has risen steadily, with 358 new treatment starts in 2025 compared to 170 in 2014.

Clinical outcomes following SSRS at our institution have been reported extensively in prior publications. For example, a retrospective study of 332 patients with spinal metastases treated at our institution between 2002 and 2012, with a median follow-up of 33 months, demonstrated a 1-year local control rate of 88% and overall survival rates of 64% [6]. Among a subset of 52 patients who survived more than four years, the 5-year local control was 91%, with grade ≥ 3 toxicity observed in only 2% of treated sites [13]. Histology-specific analyses further underscore the efficacy of SSRS: 67 patients with metastatic thyroid cancer achieved 96% 1-year local control [7], 43 patients with metastatic renal cell carcinoma had 82% [10], and 70 patients with metastatic sarcoma reached 89% [14]. Additionally, a small cohort of seven patients with metastatic pheochromocytoma showed a crude local control rate of 94% [12]. In a separate study of 38 patients receiving SSRS for oligometastatic disease, outcomes included a median overall survival of 6.3 years and a prolonged median time to systemic therapy modification of 3.4 years.

Despite its demonstrated clinical efficacy, SSRS is a technically demanding treatment modality that requires precise patient immobilization, high-quality multimodality imaging, accurate target delineation, and rigorous quality assurance. Submillimeter uncertainties in image registration, target localization, or treatment delivery can result in clinically meaningful dose deviations to the spinal cord or target volume. As SSRS programs expand in volume and complexity, maintaining consistency and safety across staff, imaging platforms, and treatment technologies becomes increasingly challenging.

**Figure 1:**
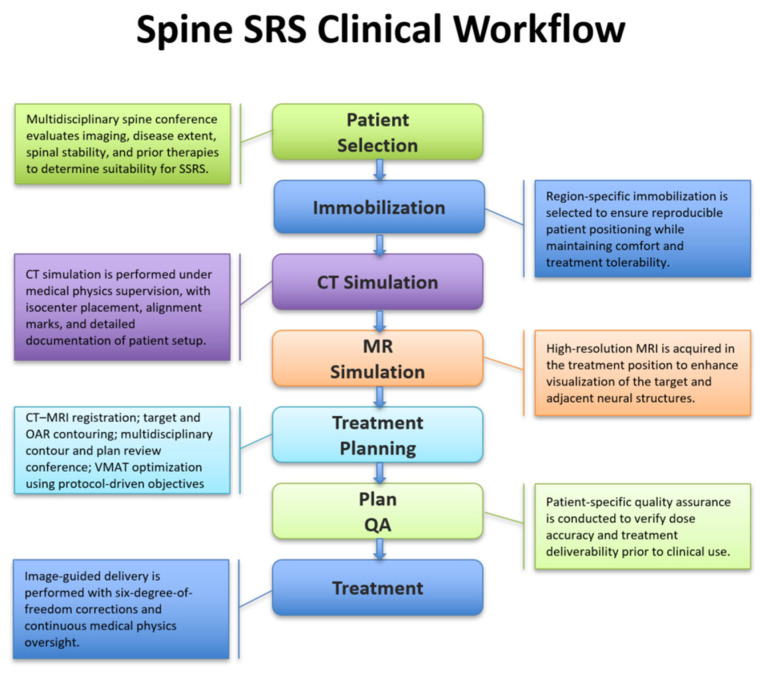
Clinical workflow for stereotactic spine radiotherapy, illustrating the sequential steps from patient selection through image-guided treatment delivery.

While numerous studies have reported clinical outcomes following SSRS, relatively few publications describe the training requirements and clinical workflows necessary to support safe and reproducible implementation, particularly in high-volume settings. The purpose of this manuscript is not to present new quantitative outcome or performance data, which have been reported previously, but rather to describe the integrated clinical workflow used at our institution. We describe staff training and credentialing, immobilization and simulation procedures, treatment planning and quality assurance processes, and image-guided treatment delivery (Figure 1), with emphasis on the medical physics responsibilities across each phase of care. This workflow-focused approach is intended to complement existing quantitative studies and to provide a practical reference for centers developing or refining spine stereotactic radiotherapy programs.

## 2. Training and Credentialing

Physician training begins with a series of educational lectures covering key topics such as patient selection, target and organ-at-risk contouring, treatment plan evaluation, and delivery techniques. As part of the credentialing process, physicians are required to complete at least one post-operative case and one case from each of the four spinal regions—cervical, thoracic, lumbar, and sacral—which are subsequently reviewed by a senior central nervous system radiation oncologist. In addition, trainees must regularly attend multidisciplinary spine tumor board meetings and actively participate in patient simulations and treatment sessions. A six-month probationary period follows, during which all contours, planning directives, and treatment plans are reviewed in detail with an experienced SSRS physician to ensure consistency and quality. A maintenance of certification process follows to ensure adequate annual clinical volumes for competency.

For physicists at our institution, credentialing for spine stereotactic radiosurgery requires full licensure by the state of Texas, successful completion of a six-month institutional probationary period, and participation in structured SSRS-specific training. This training is conducted over several months and is designed to gradually transition the physicist from observation to supervised independence. Trainees begin by observing experienced SSRS physicists during patient simulations and treatments, with particular attention to technical execution, interdisciplinary communication, patient interaction, and impromptu clinical decision-making. As competency develops, the trainee assumes increasing responsibility for clinical tasks, initially under close supervision and later with more autonomy. Throughout the process, experienced physicists remain available for guidance, ensuring both safety and consistency with departmental standards. Credentialing is granted once the physicist has actively participated in a minimum of ten SSRS treatments and receives approval from the supervising physicists and the physics section chief, confirming readiness for independent practice.

## 3. Patient Selection

One of the hallmarks of the SSRS program is strong multidisciplinary teamwork with intense involvement of neurosurgery and radiology, from patient selection to treatment planning for every patient. Patients considered for SSRS treatments are reviewed in a weekly spine conference comprising radiation oncologists, neurosurgeons, a diagnostic radiologist (either musculoskeletal or neurologic), advanced practice providers, radiation oncology residents, and medical physicists. After reviewing the images and clinical history, the team discusses treatment options, which include stereotactic or conventional radiation therapy, surgery, spine laser interstitial thermal treatment, systemic treatments, and/or observation. Indications for SSRS typically include re-irradiation, radioresistant tumor histology requiring radiotherapy, oligometastatic/oligoprogressive disease, and/or durable pain relief in well-selected patients. Contraindications for SSRS typically include greater than three involved vertebral levels, inability to tolerate simulation/treatment (e.g., uncontrolled pain), and/or inability to obtain MRI (e.g., due to MR safety concerns). Surgery is considered for spinal instability and/or for high-grade epidural spinal cord compression (i.e., Bilsky grade 2–3) to remove the epidural tumor (“separation surgery”), thereby allowing adequate dose to the target postoperatively without exceeding spinal cord constraints. Spinal laser interstitial thermal therapy is a minimally invasive alternative to separation surgery for patients who may not tolerate surgery or the associated delays in the initiation of systemic therapy. Catheters are placed adjacent to the epidural tumor with MRI guidance, allowing a laser probe to ablate epidural tumors under real-time heat monitoring, which is typically followed by SSRS within 1 week.

## 4. Immobilization

**Figure 2:**
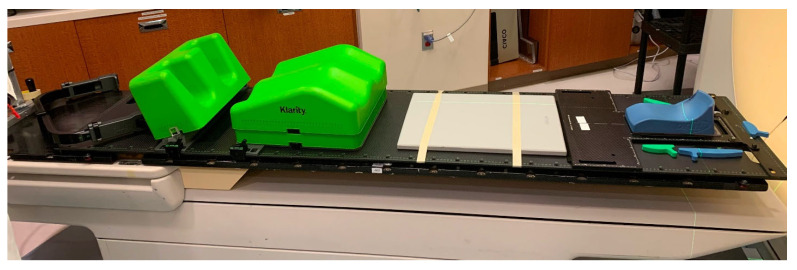
The Klarity SBRT system (Klarity Medical, Heath, OH, USA) with a modular carbon-fiber overlay and customizable components designed to streamline patient setup and maintain high immobilization precision.

Effective immobilization is essential in SSRS to enable precise treatment delivery with steep dose gradients and minimal setup uncertainties. From 2002 to 2020, our institution utilized the BodyFix^®^ system (Elekta Solutions AB, Stockholm, Sweden), a full-body vacuum cradle, for SSRS patient positioning. While this system provided excellent stability and reproducibility, it posed logistical challenges as patient volumes increased. Transporting the large cradles between facilities for MR simulation and treatment became cumbersome, and in some cases, the cradles did not fit through the MRI bore. These limitations prompted a transition in April 2020 to the Klarity SBRT system (Klarity Medical, Heath, OH, USA), which employs a modular design with smaller, adjustable components that are easier to store and transport (Figure 2). This system also reduces simulation time while maintaining patient setup reproducibility comparable to the full-body cradle [16].

**Figure 3:**
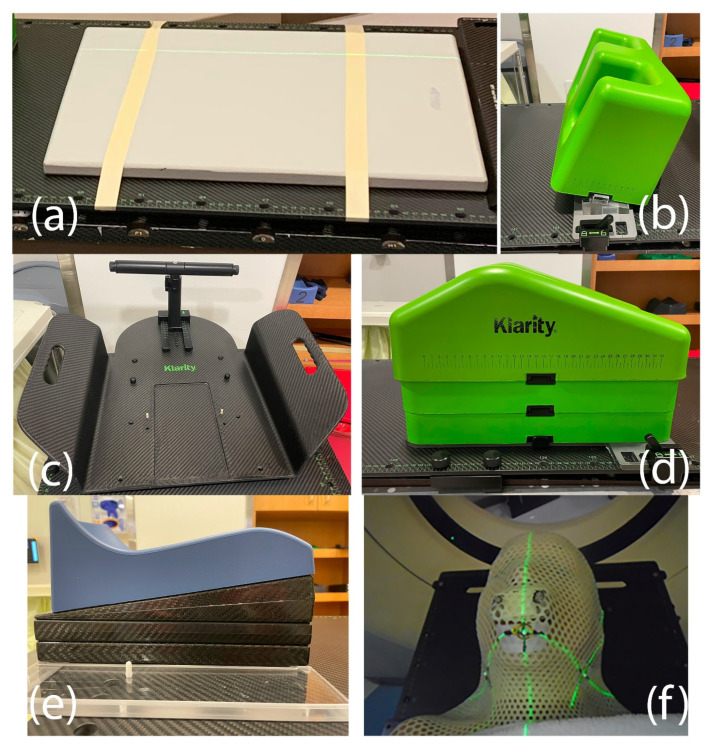
Core components of the Klarity SBRT immobilization system: (**a**) low-profile cushion (CQ Medical); (**b**) angled foot positioner; (**c**) wingboard for arm positioning; (**d**) adjustable knee cushion with blocks; (**e**) modified head cradle with positioning blocks and head cushion; (**f**) thermoplastic head-and-shoulders mask (Orfit Industries) with integrated bite block.

The core components of the Klarity SBRT immobilization system include a carbon-fiber table overlay, adjustable knee support, and a foot holder (Figure 3). A low-profile cushion (CQ Medical, Orange City, IA, USA) is used to provide patient comfort without compromising flat positioning of the spine (Figure 3d). Additional modular accessories can be incorporated as needed, allowing customization based on the spinal region being treated while maintaining reproducibility and ease of setup.

**Figure 4:**
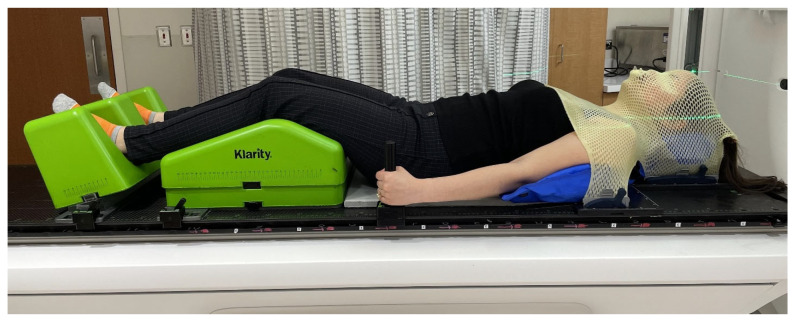
Head-and-shoulders thermoplastic mask with bite block used for upper thoracic and cervical spine immobilization. Customized hand grips support reproducible shoulder and clavicle positioning.

For upper thoracic spine and cervical spine treatments, a head-and-shoulder mask (Orfit Industries America, Norfolk, VA, USA) and a head cradle (Klarity Medical) are used to immobilize the treatment region (Figure 4). A bite block helps to maintain the chin position and head pitch during upper cervical spine treatments. Shoulder and clavicle reproducibility is achieved with customized hand grips.

**Figure 5:**
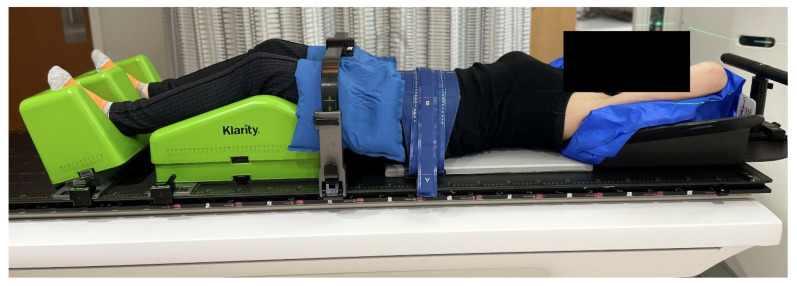
Immobilization setup for mid- and lower-thoracic spine treatments using a wingboard with vacuum-lock support. This configuration positions the patient’s arms overhead to maintain treatment clearance and reproducible upper body alignment.

**Figure 6:**
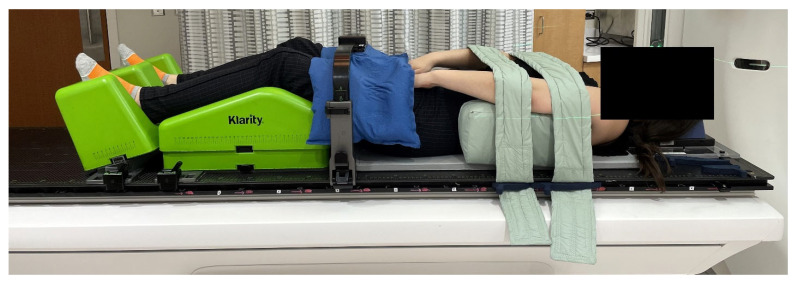
Arms-down immobilization configuration for patients unable to tolerate arms-up positioning. Foam blocks and padded straps, developed in-house, support the arms comfortably and reproducibly.

**Figure 7:**
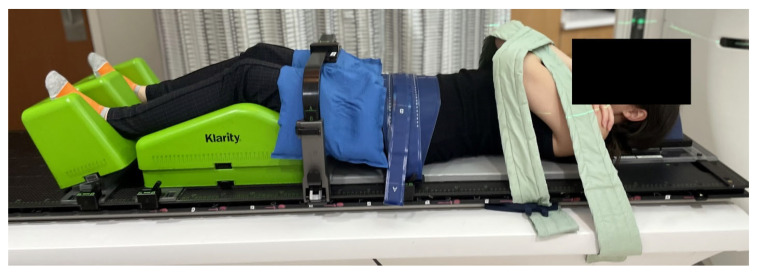
Arms-crossed setup used for lumbar and sacral spine treatments. Straps provide support while maintaining reproducibility and patient comfort.

For the mid- and lower-thoracic region (Figure 5), the patient’s arms are raised overhead using a wingboard and cradle to support the arms and shoulders. A respiratory belt is placed mid-level to maintain positioning, and a cradle is placed over the upper legs to create reproducible hip positioning. Patients who cannot tolerate raised arms are placed in an arms-down position, supported by foam blocks and padded straps developed and produced in-house (Figure 6). For the lumbar and sacral regions (Figure 7), the arms are preferably crossed and supported by straps. The respiratory belt and leg cradle are also used in this setup. As with the thoracic spine setup, patients may be alternately placed in an arms-down arrangement.

## 5. CT Simulation

Before simulation, the treating team obtains informed consent and provides education about the treatment process. The visit also addresses pain/anxiety, including anesthesia options when appropriate. Patients are asked to wear athletic clothing—e.g., pants and, when applicable, a metal-free sports bra—to maintain comfort and modesty while enabling accurate positioning for simulation and treatment.

For simulation preparation, the physicist reviews the chart and orders with attention to the treatment site, prior radiation, and relevant surgeries/hardware. Tumor location is confirmed on diagnostic MRI (with PET/CT when available). The physicist assesses lesion visibility on CT and notes anatomic variants (e.g., sacralization) that could affect positioning or image registration.

CT simulation is performed by the therapy team following institutional standard operating procedures aligned with AAPM Medical Physics Practice Guidelines [17]. A physicist is present for the entire simulation. As previously described, patient immobilization is tailored to the treatment region. A scout image is first acquired to ensure the patient is aligned and reproducibly straight. A localization scan is then performed, typically extending from the C1 vertebra to several vertebral bodies below the region of interest, or from the sacrum to several vertebral bodies above, depending on the target location.

The entire team reviews the initial images to confirm coverage of the intended treatment area. An isocenter is then selected, generally centered on the vertebral body or bodies of interest in the superior-inferior and lateral directions. The vertical coordinate is typically aligned with the vertebral body but may be placed anteriorly to facilitate the placement of alignment marks. Alignment lasers are used to position three radiopaque BBs on the patient—one anterior and two lateral—ensuring that the isocenter is visible on the planning CT scan. A second scout is acquired to verify positioning, followed by a final CT scan with 1 mm slice thickness for treatment planning. All scans are acquired on one of three Philips Brilliance Big Bore scanners with the acquisition parameters listed in Table 1.

During simulation, the physicist documents the configuration of each immobilization component, including specific settings and positions, as well as any relevant details regarding patient setup. This information is recorded in a standardized form and supplemented with photographs of the immobilization, all of which are uploaded to the record-and-verify system to instruct MR simulation and treatment delivery. To ensure continuity and consistency, the physicist who oversees the simulation also participates in treatment planning and performs the initial review of the treatment plan. CT simulations are scheduled for 45 min per setup, although most are completed within 30 min.

## 6. MR Simulation

**Figure 8:**
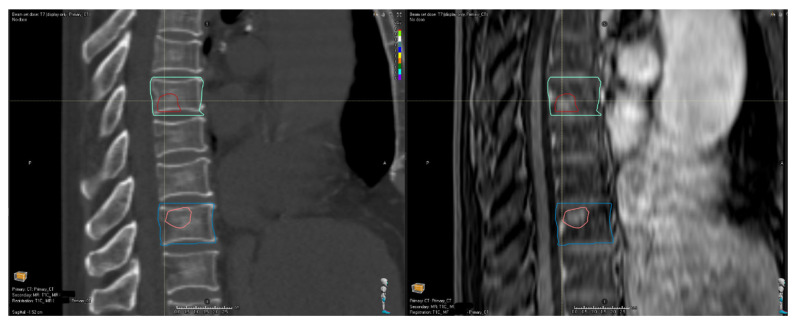
A simulation CT (**left**) and simulation MRI (**right**) co-registered to simultaneously align the 7th and 10th thoracic vertebrae.

Following CT, MR simulation provides high-contrast imaging in the treatment position, reducing curvature discrepancies seen on diagnostic scans. The limited field of view permits 1–2 mm slice thickness. Immobilization matches CT except for an MR-safe fiberglass table overlay and wingboard (Klarity) in place of carbon fiber. Patient-specific components (thermoplastic mask, bite block, head cradle, knee cradle) are reused to maintain identical positioning. Figure 8 shows co-registered MRI and CT images for a patient with isocenters at the T7 and T10 levels. Using identical immobilizations for the CT and MRI enables simultaneous registration of these levels, which are 7 cm apart.

MR simulation images are acquired using one of two scanners: a Siemens Sola Fit 1.5 T or a Siemens Vida 3.0 T (Siemens Healthineers AG, Forchheim, Germany). For cervical spine imaging, a combination of two large Flex 4 coils (used with the Sola 1.5 T scanner), two small UltraFlex 18 coils (used with the Vida 3.0 T scanner), and integrated spine coils is used, while thoracic and lumbar spine imaging is performed using an anterior Body 18 coil in conjunction with posterior spinal coils. The imaging protocol includes T2- and pre-contrast T1-weighted sequences, followed by a post-contrast T1-weighted Dixon sequence, as outlined in Table 2. Treatment is typically scheduled 6 to 7 business days after MR simulation, with timing dependent on case complexity and planning requirements.

## 7. Treatment Planning

Treatment planning is performed using RayStation^®^ (RaySearch Laboratories AB, Stockholm, Sweden). Planning begins with rigid registration of the MR images to the planning CT. To guide alignment, a temporary vertebral volume of interest is contoured to include the target vertebrae plus one level superior and inferior. Gray-level-based registration is then performed using this volume as the focal region, followed by multiplanar review and manual refinements as needed. In select cases, the CT localization scan is also imported to assist with target identification.

Each MR image set is registered independently to the planning CT using this rigid, gray-level-based approach, including rotational alignment. Frame-of-reference-based registration is not used, as patient motion between MRI acquisitions may be present. Registration accuracy is reviewed in multiple planes and is evaluated jointly by the attending physician and medical physicist during treatment planning to ensure appropriate alignment of the target vertebrae and adjacent neural structures.

The post-contrast T1-weighted sequence is primarily used for gross tumor volume (GTV) delineation, while the pre-contrast T1-weighted scan helps distinguish tumor from adjacent normal tissues. The T2-weighted sequence is used to contour critical neural structures, including the spinal cord, cauda equina, and sacral nerve roots. In patients with metal spinal fixation hardware, CT myelography—performed in diagnostic radiology—may be used to enhance visualization of the spinal cord when MR image quality is compromised.

Image registration accuracy is further supported by routine MRI quality assurance. MRI geometric distortion is evaluated annually and following major system upgrades or repairs using a three-dimensional geometric QA phantom, with submillimeter geometric accuracy verified within a 30-cm diameter spherical volume centered at isocenter. In addition, geometric fidelity is monitored routinely through daily ACR phantom testing.

**Figure 9:**
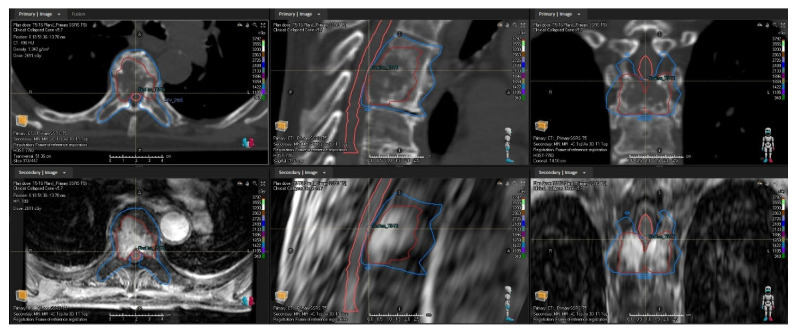
A CT (**top row**) and T1 + contrast MRI (**bottom row**) showing the GTV (dark red), CTV (blue), and spinal cord (pink). The treatment targets the 5th and 6th thoracic vertebrae.

The attending radiation oncologist contours the gross tumor volume (GTV), clinical target volume (CTV), and the organs most at risk, which may include the spinal cord, cauda equina, nerve roots, trachea, and esophagus. CTV delineation follows international consensus guidelines [18,19,20]. The dosimetrist contours the remaining critical structures. Figure 9 shows typical GTV, CTV, and spinal contours for a metastatic tumor in the 5th and 6th thoracic vertebrae.

Once contouring is complete, the physicist reviews the image registrations, contours, couch placement, and any necessary density overrides for hardware or other structures. Consistency between the prescribed target location and the contoured volume is also verified. The physicist then selects the final isocenter, and, to reduce the effects of rotational uncertainties, positions it near the center of the CTV and in proximity to the organ most at risk—typically the spinal cord or cauda equina. Volumes that fall outside the Hounsfield-unit–to–electron-density calibration range (−1000 to 3734 HU) are flagged for material overrides before dose calculation. The plan is then returned to dosimetry for treatment planning.

To promote consistency and improve efficiency, planning is initiated using a protocol implemented via the RayStation scripting interface. A graphical user interface allows entry of target and organ-at-risk (OAR) dose constraints based on the physician’s planning directive. The script then configures the dose grid, beam arrangement, planning structures, optimization objectives, and clinical goals. Once the script completes, dosimetrists refine the objectives and beam parameters to meet the specific needs of each patient. The default objectives are tailored for cases with favorable geometry—typically those with at least 6 mm of separation between the GTV and adjacent OARs. Cases with more complex geometry require additional customization by the dosimetrist. All treatment plans use 6 MV photon beams, and the dose, reported as dose to water, is calculated using a collapsed cone convolution algorithm [21,22].

A plan is considered ready for physics review when all clinically achievable objectives have been met, and further improvements would result in a cost, such as increased dose to an organ at risk (OAR) or reduced dose to the target. Treatment plans must meet the dose constraints for critical structures—particularly the spinal cord and cauda equina, which are often adjacent to the GTV. Ensuring adequate minimum dose—15 Gy and 21 Gy for single and multi-fraction treatments, respectively—to the GTV is also particularly important, as it has been associated with durable local control [6]. At least 95% coverage of the GTV and 80% coverage of the CTV are also heavily weighted. Additional planning goals include dose conformality, limiting the maximum dose to ≤120% of the prescription, and maintaining appropriate dose falloff.

Treatment plans are reviewed twice weekly in a multidisciplinary conference that includes radiation oncologists, neurosurgeons, and medical physicists. The focus of the conference is on target and critical structure delineation, image registration accuracy, and the planning directives, particularly the dose constraints assigned to organs at risk and target volumes.

Dose prescription is based on tumor histology and the patient’s prior radiation history. For radiation-naïve sites, a single fraction of 18 Gy is typically prescribed, or 24 Gy for radioresistant histologies. In these cases, the clinical target volume (CTV) is uniformly prescribed 16 Gy across all histologies. For previously irradiated sites, the standard prescription is 27 Gy to the gross tumor volume (GTV) delivered in three fractions. The CTV receives 21 Gy for radiosensitive tumors and 24 Gy for radioresistant tumors. Single-fraction regimens are generally preferred due to their association with improved local control [10,23]. The regimens are summarized in Table 3.

Planning goals include a minimum dose objective of D_0.01_cc ≥ 15 Gy to the GTV in single-fraction treatments to reduce the risk of local failure. The spinal cord is typically constrained to D_0.01_cc ≤ 12 Gy. For patients with prior spinal cord irradiation, the maximum allowable dose is dependent on the amount of prior radiation, time interval since prior RT, patient prognosis, and physician discretion. The planning goals for key organs at risk, listed in Table 4, may be modified by the attending physician on a case-by-case basis.

Most treatment plans utilize four coplanar volumetric modulated arc therapy (VMAT) beams, with gantry angles spanning 90–179° and 181–270°, which may be further reduced to avoid treating through the arms. In select cervical spine cases, the addition of anterior beams may improve target coverage and help reduce the dose to surrounding normal tissues.

The physicist evaluates the plan to confirm that it meets the goals outlined in the planning directive and is consistent with general expectations for plan quality. The radiation oncologist then reviews and approves the plan. Before proceeding to treatment preparation, the plan undergoes peer review by a second radiation oncologist credentialed in stereotactic spine treatments.

## 8. Treatment Plan Quality Assurance

Before treatment, every plan undergoes patient-specific quality assurance (PSQA). Treatment beams are exported from the treatment planning system and recalculated on a digital representation of the OCTAVIUS 4D phantom (PTW, Freiburg, Germany). The plan is then delivered and measured with the OCTAVIUS 4D system, a polystyrene cylinder housing 977 liquid-filled ionization chambers (2.5 × 2.5 × 0.5 mm^3^). Measured and calculated dose distributions are compared in VeriSoft (PTW) using 3D gamma analysis [24] with a 2% dose-difference and 2 mm distance-to-agreement criterion (local-dose); plans pass if at least 90% of evaluated points meet the criterion. Low-dose regions (<30% of the normalization value) are excluded from evaluation. Nearly all plans meet criteria on the first attempt; rare exceptions—typically highly modulated plans for larger targets—are re-optimized to reduce total monitor units and improve the gamma pass rate. This PSQA process verifies both dose accuracy and plan deliverability, including complete transfer of beam data from the treatment planning system through the record-and-verify system to the linear accelerator.

## 9. Treatment

Treatment is delivered using a Varian TrueBeam linear accelerator (Varian Medical Systems, Palo Alto, CA, USA) equipped with high-definition multileaf collimators. Prior to each treatment, radiation therapists configure the immobilization devices, and the physicist verifies the setup. The patient is positioned on the Klarity overlay and initially aligned to the marked isocenter from the simulation. Additional immobilization components are then placed, and the treatment couch is used to shift the patient to the final isocenter established during treatment planning.

X-ray images are acquired using ExacTrac Dynamic (Brainlab AG, Munich, Germany) and registered to the digitally reconstructed radiographs (DRRs) generated from the planning CT. Using six degrees of couch freedom, positional corrections from the ExacTrac registration are applied to bring the patient into treatment position. Applying 6D couch pitch and roll corrections reduces position-dependent setup error that otherwise increases with distance from isocenter. Once alignment is within our institutional tolerance (≤0.7 mm and 0.8°), cone beam CT (CBCT) and orthogonal kV/MV images are acquired for verification. CBCT allows visualization of the spinal canal, while the planar images provide confirmation of gross anatomical alignment to ensure the correct vertebral body is targeted. Final alignment is verified by the attending physician in accordance with national guidelines [25]. The physicist provides continuous supervision throughout the procedure [18], reviewing patient positioning and assisting with troubleshooting as needed.

Before treatment can begin, the physician must approve the CBCT, kV/MV pair, and ExacTrac images within the record-and-verify system. Immediately prior to beam-on, the therapists acquire a final set of ExacTrac images to confirm that the patient has not shifted. Treatment proceeds only after all team members agree that alignment is satisfactory. Additional ExacTrac images are acquired prior to each arc or whenever there is concern about potential patient movement to verify the patient position remains within tolerance. Treatments are scheduled for 40 min but may take longer if the patient setup is difficult to reproduce.

## 10. Discussion

This manuscript describes the clinical workflow and medical physics processes used to support spine stereotactic radiotherapy at a high-volume academic center. Unlike prior publications from our group that have focused on quantitative evaluation of clinical outcomes, dosimetric performance, and technical accuracy, the present work is intended to document how these validated techniques are integrated into routine clinical practice. By detailing training, simulation, planning, quality assurance, and image-guided delivery, this manuscript aims to provide practical insight into the operational requirements of a mature SSRS program.

Stereotactic spine radiotherapy continues to advance. In the near term, workflows like those described here are likely to remain standard, with MR-guided and CBCT-guided adaptive techniques representing the next step. MR-guided adaptive therapy offers superior soft-tissue visualization for both target and cord, enabling on-table plan adaptation to enhance coverage while respecting cord constraints [26,27,28]. Cunningham et al. demonstrated online adaptive planning and intrafraction management on an MR-linac for spine SBRT, improving cord localization and permitting dose redistribution to the target [27]. Using an adapt-to-shape workflow on the MR-linac, Han et al. reported increases in minimum GTV dose of approximately 10–50% with only small increases in cord dose (~3%) in a re-irradiation setting [28]. In parallel, increasing automation in contouring and planning is compressing the interval from simulation to treatment to hours rather than days. Advances in CBCT image quality now support CBCT-based online adaptation or simulation-free workflows using prior plan libraries [29].

The favorable local control and toxicity outcomes cited in this manuscript are derived primarily from historical institutional series spanning multiple eras of SSRS implementation. While these studies demonstrate the overall effectiveness of spine stereotactic radiotherapy and radiosurgery at our institution, they do not represent a prospective validation of the exact workflow configuration described here. Over time, substantial changes have occurred in immobilization systems, imaging protocols, treatment planning software, image guidance platforms, and quality assurance processes. Consequently, the reported clinical outcomes should be interpreted as supporting the SSRS approach in general, rather than as outcomes directly attributable to the current workflow in its present form. In addition, patients treated within this program represent a highly selected population evaluated through a multidisciplinary spine conference, which may further limit the generalizability of both outcomes and workflow feasibility to broader clinical settings.

The workflow described is tightly integrated with a specific set of commercial technologies, including RayStation treatment planning with a collapsed cone convolution dose calculation algorithm, Varian TrueBeam linear accelerators with high-definition multileaf collimators, ExacTrac Dynamic for six-degree-of-freedom image guidance, and Octavius 4D-based patient-specific quality assurance. While this integrated platform supports a high degree of precision and reproducibility, it may limit direct applicability to centers using alternative planning systems, dose calculation engines, image guidance strategies, or QA methodologies. Differences in imaging quality, registration tools, motion management capabilities, and automation infrastructure may influence achievable setup accuracy, plan quality, and workflow efficiency. As a result, centers without access to similar technologies or staffing models may require workflow modifications or additional validation to achieve comparable performance.

## 11. Conclusions

This manuscript describes the clinical workflow used to deliver spine stereotactic radiotherapy at a high-volume academic center, with emphasis on the medical physics processes that support routine clinical practice. By documenting the integration of training, simulation, treatment planning, quality assurance, and image-guided delivery, this work complements previously published quantitative studies and provides a descriptive account of how spine stereotactic radiotherapy is implemented in an established clinical program.

## Figures and Tables

**Table 1:** Imaging settings used for CT simulation.

Manufacturer	Model	kVp	mAs	Convolution Kernel	Reconstruction FOV (mm)	Slice Thickness (mm)	Rows	Columns
Philips	Brilliance Big Bore	120	400	B	600	1.0	512	512

**Table 2:** Imaging settings used for MR simulation.

MR Scanner	Siemens Sola Fit 1.5T			Siemens Vida 3.0T		
Sequence Type	T2	T1	T1 + Contrast	T2	T1	T1 + Contrast
Acquisition Type	3D Spin-echo	3D Gradient-echo	3D Gradient-echo	3D Spin-echo	3D Gradient-echo	3D Gradient-echo
Fat Saturation	No	No	Dixon	No	No	Dixon
TE/TR (ms)	150/1200	4.76/7.4	2.4/4.8/7.11	123/1200	2.46/4.96	1.34/2.53/4.25
FOV (cm^3^)	220 × 220 × 176	220 × 220 × 176	220 × 220 × 176	220 × 220 × 160	220 × 220 × 160	300 × 300 × 160
Voxel Size (mm^3^)	0.43 × 0.43 × 1	0.43 × 0.43 × 2	0.43 × 0.43 × 2	0.43 × 0.43 × 1	0.43 × 0.43 × 2	0.6 × 0.6 × 1
Pixel Bandwidth (Hz)	300	400	407	445	440	1150
Flip Angle (deg)	150	12	10	120	12	10
Scan Time (min)	4:44	7:28	5:20	5:53	3:40	6:00

**Table 3:** Spine Stereotactic Radiation Therapy Dose Prescriptions.

Prior Radiation	Histology	GTV Dose	CTV Dose	Number of Fractions
No	Radiosensitive	18 Gy	16 Gy	1
No	Radioresistant	24 Gy	16 Gy	1
Yes	Radiosensitive	27 Gy	21 Gy	3
Yes	Radioresistant	27 Gy	24 Gy	3

**Table 4:** Planning goals for organs at risk.

Organ at Risk	Single Fraction	Three Fraction
Spinal Cord	Volume receiving 10 Gy < 1 cc	Volume receiving 9 Gy < 1 cc
	Maximum to 0.01 cc < 12 Gy	Maximum to 0.01 cc < 10 Gy
Cauda Equina	Volume receiving 14 Gy < 1 cc	Volume receiving 12 Gy < 1 cc
	Maximum to 0.01 cc < 16 Gy	Maximum to 0.01 cc < 14 Gy
Esophagus	Volume receiving 14 Gy < 1 cc	Volume receiving 14 Gy < 1 cc
	Maximum to 0.01 cc < 17 Gy	Maximum to 0.01 cc < 21 Gy
Brachial Plexus	Volume receiving 14 Gy < 3 cc	Volume receiving 15 Gy < 0.1 cc
	Maximum to 0.01 cc < 16 Gy	Maximum to 0.01 cc < 18 Gy
Heart	Volume receiving 16 Gy < 15 cc	Volume receiving 15 Gy < 15 cc
	22 Gy Maximum	21 Gy Maximum
Trachea	Volume receiving 8.8 Gy < 4 cc	Volume receiving 8.8 Gy < 4 cc
	20.2 Gy Maximum	18 Gy Maximum
Skin	Volume receiving 14 Gy < 10 cc	Volume receiving 16 Gy < 10 cc
	16 Gy Maximum	21 Gy Maximum
Small Bowel	Volume receiving 10 Gy < 5 cc	Volume receiving 12 Gy < 0.01 cc
	15.4 Gy Maximum; Circumferential Dose < 12 Gy	Circumferential Dose < 10 Gy
Colon	Volume receiving 11 Gy < 20 cc	Volume receiving 11 Gy < 20 cc
	18.4 Gy Maximum	18 Gy Maximum
Sacral Plexus	Maximum less than GTV prescription dose	Maximum less than GTV prescription dose

## Data Availability

No new data were created or analyzed in this study. Data sharing is not applicable to this article.
